# Burnout syndrome in Spanish medical students

**DOI:** 10.1186/s12909-021-02661-4

**Published:** 2021-04-22

**Authors:** Javier Gil-Calderón, Jéssica Alonso-Molero, Trinidad Dierssen-Sotos, Inés Gómez-Acebo, Javier Llorca

**Affiliations:** 1 Universidad de Cantabria, Santander, Spain; 2 Universidad de Cantabria – IDIVAL, Avda. Herrera Oria s/n, 39011 Santander, Cantabria Spain; 3 CIBER Epidemiología y Salud Pública (CIBERESP), Madrid, Spain

**Keywords:** Burnout, Medical student, Family support, Maslach burnout inventory

## Abstract

**Background:**

Burnout syndrome is a frequent syndrome related to people that feel a deterioration in their daily activities due to highly demandant psychological requirements in their workplaces. Within last decades, this syndrome has been studied across medical professionals, concluding that stress levels that physicians suffer is high enough to make them develop burnout syndrome. In the case of medical students, there are some recent studies, although with small samples. For this reason, given that this phenomenon may produce a huge impact in medical students’ development, the aim of this study is to analyze the influential factors that may contribute to its occurrence.

**Methods:**

The necessary information was gathered through a web-based questionnaire, divided in two parts. The first part of the survey included questions related to personal aspects of the students. Burnout related questions (second part) were divided in three subscales to evaluate exhaustion, cynicism, and academic efficacy levels.

**Results:**

Family support for studying medicine is associated with lower burnout levels in all three scales of the Maslach Burnout Inventory. The number of years spent in the degree show the opposite trend: the more years in the degree, the higher score in all burnout scales.

**Conclusions:**

Burnout syndrome is a problem among medical students in Spain that increases with the number of years studying medicine. It should be also noticed that family support and vocational studies are independent factors related to lower levels of burnout.

**Supplementary Information:**

The online version contains supplementary material available at 10.1186/s12909-021-02661-4.

## Introduction

Burnout syndrome is increasingly frequent and is related to people that suffer a deterioration in their daily activities due to highly demandant psychological requirements in their workplaces [1]. Freudenberger described burnout syndrome as the stress suffered by those people who work in contact with other people [2]. Maslach and Jackson gave its definitive definition as a syndrome of emotional exhaustion and cynicism that occurs frequently among individuals who do “people-work” of some kind [3].

Within last decades, burnout syndrome has been studied across medical professionals, concluding that physicians suffer stress levels high enough to make them develop it [1, 4, 5]. In the last years, attention has also been paid to the presence of the burnout syndrome in medical students [6–9]. However, most of these studies have relatively small student participation [8, 9], which makes it necessary to increase research in this field.

When it comes to speak about students, burnout syndrome is defined as lack of concentration, inability to focus, difficulty in retaining information, experiencing recurrent headaches, lack of sleep, feeling fatigued and helpless, not putting up the best efforts, and experiencing unknown hesitation due to academic stressors and performance anxiety [6]. Students, especially those that are enrolled at University, are frequently immersed in situations, activities and academic events that generate stress and anxiety, like compulsory presentations, lack of time and task overload [3]. In such way, when the exposition to the stressors is produced habitually and students lack strategies to confront it properly, academic burnout syndrome may appear [3]. These students would show high levels of emotional tiredness or exhaustion, cynicism about their studies, and low efficacy in the development of academic activities [3].

Maslach Burnout Inventory (MBI; Maslach y Jackson, 1986) [3] is the validated survey to test the burnout syndrome in health care professional, whereas Schaufeli, Martínez, Pinto, y Salanova, 2002 adapted it for students [7]. Their questionnaire tests the feelings and attitudes of students to their academic activity, using 15 items grouped into three subscales (emotional exhaustion, depersonalization and academic efficacy).

Given that this phenomenon may produce a huge impact in medical students’ development, it is relevant to study the risk factors of this syndrome within this collective, in order to develop strategies that may improve the symptoms and reduce the consequences. Having explained that and knowing the fact that the search developed until now contemplates comparatively small samples, the aim of this study is to analyze the influential factors that may contribute to the presence of burnout symptoms in medical students in a sample of 1073 medicine students.

## Methods

### Setting and participants

This project was designed as a cross sectional study based on the results obtained from the MBI-SS (Maslach Burnout Inventory-Student Survey) conducted between April and May of 2019, given to medical students from all over Spain. The survey was targeted to medical students from the first year to the last one (in Spain, the Medicine Degree lasts for 6 years).

The target population was reached by sending the survey to the CEEM (Spanish Medical Students Council), that distributed it within the Medical Faculties in Spain. Student representants of the Faculties do not participating in CEEM were independently contacted to distribute the survey. Students from 32 out of the 42 Medical Faculties in Spain were reached and filled the survey.

The only inclusion criterium was to be a medical student appointed in any Medical School in Spain, without any exclusion criteria.

### Gathered information

The information was gathered through a web-based questionnaire including questions about the personal situation of the participants and the MBI-SS [7]. This survey was anonymous, and the complete survey employed is in the Additional files 1 and 2.

The first part of the survey included questions related to personal aspects of the students as gender, age, vocation for medicine when entering in the Faculty (yes / no), family support for studying medicine, (i.e. how the students feel that their families back up that they are studying medicine. In a Likert scale from 1 -no support- to 10 -maximum support), year of the medical degree, number of years since entering in the Medicine Faculty (detailed in Additional file 1).

Burnout related questions were divided in three subscales which contained several questions each, as MBI-SS recommend. These three subscales evaluate exhaustion, cynicism and academic efficacy levels, and their questions (detailed in Additional file 2) were meant to be answered from 1 (completely disagree) to 10 (completely agree) according with the degree of agreement with the affirmations given.

### Ethical issues

The research was approved by the University of Cantabria Ethics Committee (code: CE TFG 03/2019) and was conducted according with the Spanish laws on medical research, the European Union regulations on general data protection of natural persons with regard to the processing of personal data and the Declaration of Helsinki. Regulation (EU) 2016/679 of the European Parliament and of the Council of 27 April 2016 on the protection of natural persons with regard to the processing of personal data and on the free movement of such data, and repealing Directive 95/46/EC. Available at https://op.europa.eu/en/publication-detail/-/publication/3e485e15-11bd-11e6-ba9a-01aa75ed71a1/ (accessed 04/10/19). All participants signed an informed consent.

### Statistical analysis

Firstly, all questions in the Maslach Burnout Inventory were rescaled to 1–7 in order to make them comparable with the original questionnaire. Questions related to academic efficacy were inverted as they were positive, whereas the affirmations in the other subscales were negative. After inverting academic efficacy subscale, all questions scored in the same way: the higher the scale, the higher the burn-out. Then, three scores were created by adding up the punctuation scored in each question of each subscale: (1) Exhaustion score (how tired students feel during the development of their daily academic activities); it includes 5 questions so it could score from 5 to 40. (2) Depersonalization score (how sceptic students feel about the importance of their studies); it includes 4 questions, so it could score from 4 to 32. And (3) academic efficacy score (how effective students feel developing their daily academic activities); it is obtained by adding the answers to 6 questions; it could score from 6 to 48.

Student t test was used to relate the burnout levels with gender and vocation, whereas ANOVA test was used to relate the burnout level with age, year of the degree, number of years in the degree and family support. Finally, multiple linear regression models were used to analyze which of these factors influence the score in each burnout subscale.

The statistical analysis was carried out with the software Stata/SE 16.

## Results

One thousand and seventy-three students answered the survey. Descriptive data relating to personal information can be found in Additional file 3. Almost 75% participants were women, nearly 88% participants felt vocation for medical studies, and 85% participants felt high family support for studying medicine. About 86% students were less than 24 years old, most people (63%) who answered the survey were between third and fifth year and 87% students answering the survey had been studying medicine for 5 years or less.

The five medical schools with more answers to the survey were the University of Cantabria (*n* = 197), the University of Alcala (*n* = 173), the University of Navarra (*n* = 154), the University of Barcelona (*n* = 86), and the University of Granada (*n* = 72).

Burnout subscales means±standard deviations were 14.8 ± 7.1 for depersonalization, 27.5 ± 7.2 for exhaustion and 22.4 ± 6.9 for academic efficacy, as displayed in Table 1. They were all three positively correlated with each other (Additional file 4).

**Table 1:** Results of burnout scales

	Mean ± sd	Median (IQR)
Depersonalization	14.83 ± 7.09	14.1 (8.67–19.56)
Exhaustion	27.50 ± 7.16	28.3 (22.89–33.00)
Academic Efficacy	22.38 ± 6.89	22.3 (17.67–26.22)

Table 2 displays the association between burnout scales and categorical variables. Women reported higher levels of exhaustion, while students declaring to study medicine due to vocation reported lower levels of burnout in depersonalization and academic efficacy scales. The year of the medical degree correlated positively with higher levels of exhaustion and depersonalization, and negatively with academic efficacy levels.

**Table 2:** Categorical variables associated with burnout levels

Variable	Category	n	Burnout Levels					
			Exhaustion		Depersonalization		Academic Efficacy	
			Mean ± Sd	p	Mean ± Sd	p	Mean ± Sd	p
Sex	Male	266	22.8 ± 6.7	< 0.001	13.6 ± 6.6	0.07	19.2 ± 6.6	0.25
	Female	804	24.4 ± 6.0		12.8 ± 6.0		19.7 ± 5.9	
Vocation	No	130	24.7 ± 6.2	0.27	14.6 ± 6.5	0.001	21.3 ± 6.4	< 0.001
	Yes	943	24.0 ± 6.3		12.8 ± 6.1		19.3 ± 6.5	
Year of the medical degree	1	167	22.3 ± 6.1	< 0.001	10.0 ± 5.2	< 0.001	18.6 ± 5.6	0.002
	2	123	22.7 ± 6.2		11.9 ± 6.0		19.5 ± 6.6	
	3	225	23.7 ± 6.0		12.0 ± 5.6		18.7 ± 5.8	
	4	200	24.7 ± 6.2		14.1 ± 6.4		20.7 ± 5.9	
	5	254	25.4 ± 6.1		14.6 ± 6.4		20.3 ± 6.1	
	6	104	25.0 ± 6.5		15.1 ± 6.2		19.6 ± 6.2	

**Fig. 1:**
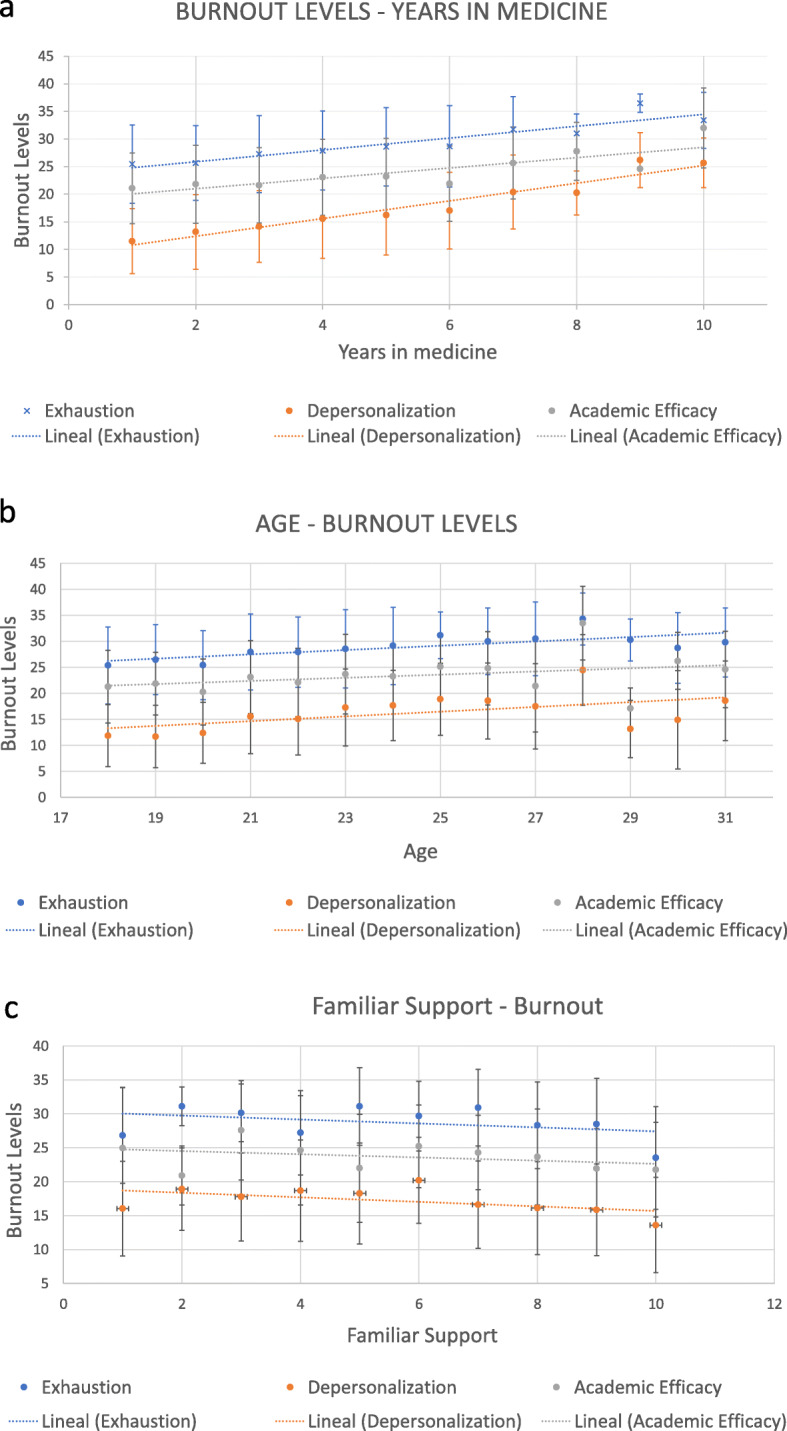
Association between burnout scales and number of years studying the medical degree (**a**), age (**b**) and family support (**c**)

Age and number of years studying the medical degree were related to higher levels of burnout in the three scores (Fig. 1a and b, respectively), and family support protects against higher levels of burnout in the three subscales (Fig. 1c).

Table 3 shows the results of the multiple regression carried out between the personal variables and the three subscales of burnout. Relating to exhaustion subscale, women scored 2.33 points more on average (95% CI: 1.23–3.44, *p* <  0.001), each year studying medicine increased exhaustion by 1.20 points (95% CI: 0.39–2.01, *p* = 0.004) and family support for studying medicine decreased exhaustion by 0.4 points (95% CI: − 0.64 - -0.15, *p* = 0.002). The remaining variables (age, year of the degree and vocation when entering the degree) were far from statistically significant.

**Table 3:** Factors that influence exhaustion, depersonalization, and academic efficacy subscales

	Exhaustion				Depersonalization				Academic efficacy			
	Coef.	95% CI		***p*** value	Coef.	95% CI		***p*** value	Coef.	95% CI		***p*** value
**Age**	0.15	−0.17	0.46	0.36	0.16	−0.15	0.46	0.31	0.09	−0.21	0.40	0.54
**Woman**	2.33	1.23	3.44	< 0.001	−0.59	− 1.65	0.46	0.27	0.90	−0.16	1.98	0.10
**Year of the degree**	−0.56	−1.38	0.27	0.19	−0.64	−1.43	0.15	0.11	−1.60	−2.45	−0.85	< 0.001
**Years studying Medicine**	1.20	0.39	2.01	0.004	1.64	0.87	2.41	< 0.001	1.78	1.01	2.56	< 0.001
**Family Support**	−0.4	−0.64	−0.15	0.002	−0.59	−0.83	− 0.35	< 0.001	− 0.46	−0.72	− 0.24	< 0.001
**Vocation**	−0.88	−2.35	0.58	0.24	−2.09	−3.49	−0.69	0.004	−2.74	−4.10	−1.27	< 0.001

Number of years studying medicine was also associated with higher levels of depersonalization burnout (1.64 points more per year, 95% CI: 0.87–2.41, *p* <  0.001), while family support (− 0.59 points, 95% CI: − 0.83 - -0.35, p <  0.001) and vocational studies (− 2.09 points, 95% CI: − 3.49 – − 0.69, p = 0.004) decreased depersonalization.

Regarding academic efficacy burnout, number of years studying medicine was the only factor associated with higher burnout level (1.78 points per year, 95% CI: 1.01–2.56, p <  0.001) while year of the degree (− 1.60 points per year, 95% CI: − 2.45 - -0.85, *p* < 0.001), family support (− 0.46 points, 95% CI: − 0.72 - -0.24, p < 0.001) and vocational studies (− 2.74 points, 95% CI: − 4.10 – − 1.27, p < 0.001) were related to lower levels of burnout.

## Discussion

One of the main results in our study was that the family support for studying medicine is associated with lower burnout levels in all three scales of the Maslach Burnout Inventory, exhaustion, depersonalization and academic efficacy, provoking students that feel higher levels of family support to suffer lower burnout levels. This hypothesis is strengthened by Santen et al. [9] who explain that it is because of family closeness is considered a protective factor that lowers stress and increases happiness. In addition, Santen et al. consider that a good ambient at home, outside of school, helps to the student get away from intense study, evaluations and, the contact with poor prognostic patients [9]. Several studies have also reported that social isolation and vulnerability worsen burnout syndrome, and that strong family relation and a correct motivation provided by relatives ease medical student’s performance and protect them [10, 11].

On the other hand, the number of years spent in the degree show the opposite trend: the more years in the degree, the higher score in all burnout scales. Previous studies have suggested that burnout levels increased each year of the medical degree [8, 12–15] although those results were not adjusted for the number of years in the degree. In addition, Galán et al. [8] found that the risk of burnout doubled from third year to sixth year in a sample of 270 Spanish medical students.

These results agree with the ones obtained in a multicentric study carried out in Chile among 1395 medical students that showed that 1 out of 2 students suffers burnout syndrome during the degree [16]. Also it is in agreement with Santen et al. results [9], where the authors affirm that one-third of all medical student show a moderate or high degree of burnout, being 43% of the third-year class. These higher levels of burnout during the development of the medical degree are associated to lower interest for the medical profession [17], which may lead to worse medical performance associated to lower levels of empathy among future physicians [18, 19] that finally leads to worsen medical performance [20]. This is one of the reasons why it has been suggested that burnout in physicians is rooted in their formative period [21].

This study has some limitations such as comparing results about burnout in the literature is not easy mainly due to the difference in the curriculum between the Schools of Medicine (e.g., age at matriculation or number of years studying medicine). Secondly, Bianchi, Schonfeld and Laurent have criticized prevalence estimations for burnout syndrome as Maslach Burnout Inventory scales have no established thresholds for diagnosing burnout [22–24]; therefore, we have avoided any cut-off and present our results in a quantitative scale. However, this procedure makes it difficult any comparison with other studies. Thirdly, about 75% participants in our study were women, which highly resembles the current sex distribution of medical students in Spanish universities; although according to our and other results, gender seems not to have association to burnout subscales [8], it is problematic to reach a define conclusion on burnout in men, given the relatively small sample of males analyzed. Finally, notice that some authors have questioned the validity of the MBI to assess the burnout syndrome in medical student [22, 25]. However, there are many authors who support its validity [26–28] and Dyrbye et al. considered MBI as “the reference standard for measuring symptoms of burnout” [29]. In fact, MBI is the most applied tool in the worldwide to test this syndrome, being used in more than 90% of all empirical studies [25].

## Conclusions

Concluding, the results of this article suggest that burnout syndrome is a problem among medical students in Spain that increases with the number of years studying medicine. It should be also noticed that family support and vocational studies are independent factors related to lower levels of burnout.

## Supplementary Information

**Additional file 1.** “Personal questions of the survey” and contain the first part of the survey.

**Additional file 2.** “Burnout questionnaire subscales and questions” and contain the second part of the survey, specific Burnout questionnaire.

**Additional file 3.** “Sample description” and contain a table whit the sample description.

**Additional file 4.** “Pearson’s correlation coefficient between the burnout scales.” and contain a table whit Pearson’s correlation coefficient between the burnout scales.

## Data Availability

The datasets generated and/or analysed during the current study are available from the corresponding author on reasonable request.
