# Supplementary material for: Burnout syndrome in Spanish medical students
Source: BMC Med Educ. 2021 Apr 22;21:231. doi: 10.1186/s12909-021-02661-4 (PMC8063293; doi:10.1186/s12909-021-02661-4)
Supplement: Supplementary file 1 — Additional file 1. “Personal questions of the survey” and contain the first part of the survey. [file 12909_2021_2661_MOESM1_ESM.pdf]

ADDITIONAL FILE 1:

TABLE 1: *Personal questions of the survey*

QUESTIONS RELATED TO PERSONAL INFORMATION
1. In which University are you studying?
2. What year of the Medicine degree are you studying?
3. How many years have you been studying the Medicine degree?
4. How old are you?
5. Did you start studying Medicine because of your vocation?
6. Do you feel familiar support to study Medicine?
7. What is your gender?
