# Supplementary material for: Burnout syndrome in Spanish medical students
Source: BMC Med Educ. 2021 Apr 22;21:231. doi: 10.1186/s12909-021-02661-4 (PMC8063293; doi:10.1186/s12909-021-02661-4)
Supplement: Supplementary file 2 — Additional file 2. “Burnout questionnaire subscales and questions” and contain the second part of the survey, specific Burnout questionnaire. [file 12909_2021_2661_MOESM2_ESM.pdf]

ADDITIONAL FILE 2:

TABLE 2: *Burnout questionnaire subscales and questions*

QUESTIONS RELATED TO BURNOUT SYMPTOMS	
EMOTIONAL EXHAUSTION	I feel emotionally drained by my studies.
	I feel used up at the end of the day at University.
	I feel tired when I get up in the morning and I have to face another day at the University.
	Studying or attending a class is really a strain for me.
	I feel burned out form my studies.
DEPERSONALIZATION	I have become less interested in my studies since my enrollment at the University.
	I have become less enthusiastic about my studies.
	I have become more cynical about the potential usefulness of my studies.
	I doubt the significance of my studies.
ACADEMIC EFFICACY	I can effectively solve the problems that arise in my studies.
	I believe that I make an effective contribution to the classes that I attend.
	In my opinion, I am a good student.
	I feel stimulated when I achieve my study goals.
	I have learned many interesting things during the course of my studies.
	During the class I feel confident that I am effective in getting things done.
