# Supplementary material for: Burnout syndrome in Spanish medical students
Source: BMC Med Educ. 2021 Apr 22;21:231. doi: 10.1186/s12909-021-02661-4 (PMC8063293; doi:10.1186/s12909-021-02661-4)
Supplement: Supplementary file 3 — Additional file 3. “Sample description” and contain a table whit the sample description. [file 12909_2021_2661_MOESM3_ESM.pdf]

ADDITIONAL FILE 4:

TABLE 4: *Sample description*

**MEDICAL STUDENTS *(1073)***			
		n	***%***
**GENDER**	Male	266	24.79%
	Female	804	74.93%
	Others	3	0.28%
**AGE**	18	114	10.62%
	19	99	9.23%
	20	163	15.19%
	21	186	17.33%
	22	197	18.36%
	23	166	15.47%
	24	68	6.34%
	25	27	2.52%
	26	17	1.58%
	27	9	0.84%
	28	3	0.28%
	29	4	0.37%
	30	6	0.56%
	>30	14	1.30%
**YEAR**	1	167	15.56%
	2	123	11.46%
	3	225	20.97%
	4	200	18.64%
	5	254	23.67%
	6	104	9.69%

**MEDICAL STUDENTS *(1073)***			
		n	***%***
**YEARS STUDYING MEDICINE**	1	185	17.24%
	2	115	10.72%
	3	211	19.66%
	4	192	17.89%
	5	230	21.44%
	6	99	9.23%
	7	23	2.14%
	8	9	0.84%
	9	2	0.19%
	>10	7	0.65%
**VOCATION**	Yes	943	87.88%
	No	130	12.12%
**FAMILY SUPPORT**	High >7	914	85.18%
	Medium	132	12.30%
	Low <3	27	2.52%
**GEOGRAPHIC UBICATION *(considering North over Extremadura, Madrid and CLM)***	Madrid	227	21.16%
	Cataluña	116	10.81%
	Islands	8	0.75%
	North	497	46.32%
	South	225	20.97%

	**University**	**n**	**%**
**UNIVERSITY**	University of Alcalá	173	16.12%
	Alfonso X el Sabio University	1	0.09%
	Autonomous University of Barcelona	12	1.12%
	Autonomous University of Madrid	18	1.68%
	University of Barcelona	86	8.01%
	University of Cádiz	15	1.40%
	University of Cantabria	197	18.36%
	Carlos the Tirth University of Madrid	0	0.00%
	University of Castilla la Mancha - Albacete	8	0.75%
	University of Castilla la Mancha – Ciudad Real	2	0.19%
	Catholic University of Valencia	0	0.00%
	CEU Cardenal Herrera	19	1.77%
	CEU San Pablo	0	0.00%
	Complutense University of Madrid	7	0.65%
	University of Córdoba	19	1.77%
	European University of Madrid	8	0.75%
	University of Extremadura	24	2.24%
	Francisco of Vitoria University	14	1.30%
	University of Girona	1	0.09%
	University of Granada	72	6.71%
	International University of Cataluña	0	0.00%

	University of Balear Islands	2	0.19%
	Jaime the First University	0	0.00%
	University of La Laguna	6	0.56%
	University of Palma de Gran Canaria	0	0.00%
	University of Lleida	0	0.00%
	University of Málaga	38	3.54%
	Miguel Hernández University	0	0.00%
	University of Murcia	3	0.28%
	University of Navarra	154	14.35%
	University of Oviedo	40	3.73%
	University of the Vasque Country	24	2.24%
	Pompeu i Fabra University	0	0.00%
	Rey Juan Carlos University	6	0.56%
	Rovira i Virgili University	17	1.58%
	University of Salamanca	30	2.80%
	University of Santiago de Compostela	24	2.24%
	University of Sevilla	13	1.21%
	University of Valencia	12	1.12%
	University of Valladolid	1	0.09%
	University of Vic	0	0.00%
	University of Zaragoza	27	2.52%
