# Supplementary material for: Burnout syndrome in Spanish medical students
Source: BMC Med Educ. 2021 Apr 22;21:231. doi: 10.1186/s12909-021-02661-4 (PMC8063293; doi:10.1186/s12909-021-02661-4)
Supplement: Supplementary file 4 — Additional file 4. “Pearson’s correlation coefficient between the burnout scales.” and contain a table whit Pearson’s correlation coefficient between the burnout scales. [file 12909_2021_2661_MOESM4_ESM.pdf]

***ADDITIONAL FILE 5:***

***TABLE 5:*** *Pearson´s correlation coefficient between the burnout scales.*

| | **Exhaustion** | **Depersonalization** | **Academic Efficacy** |
|---|---|---|---|
| **Exhaustion** | 1.0000 | | |
| **Depersonalization** | 0.5343 | 1.0000 | |
| **Academic efficacy** | 0.3640 | 0.4688 | 1.0000 |
